# The Evolving Digital Landscape of Gastroenterology Fellowship Recruitment

**DOI:** 10.14309/crj.0000000000002283

**Published:** 2026-08-12

**Authors:** Janak Bahirwani, Madhav Changela, Pravin Kalyankar, Maulik Kaneriya, Ashish Sharma, Hareesha Rishab Bharadwaj, Hassam Ali, Dushyant Singh Dahiya

**Affiliations:** 1 Department of Gastroenterology, Kadlec Regional Medical Center, Richland, WA; 2 Elson S. Floyd College of Medicine at Washington State University, Richland, WA; 3 Department of Medicine, One Brooklyn Health/Interfaith Medical Center, New York, NY; 4 Department of Internal Medicine, Yale New Haven Hospital, New Haven, CT; 5 Department of Internal Medicine, Royal Stoke University Hospital, University Hospitals of North Midlands NHS, Stoke-on-Trent, UK; 6 Department of Gastroenterology, Hepatology and Nutrition, East Carolina University/Brody School of Medicine, Greenville, NC; 7 Division of Gastroenterology, Hepatology & Motility, The University of Kansas School of Medicine, Kansas City, Kansas

**Keywords:** gastroenterology, hepatology, training, medical education, virtual reality

## Abstract

Social media has rapidly transformed communication, education, and professional networking within graduate medical education. Increasingly, residency and fellowship applicants rely on social media platforms to evaluate training programs, particularly to gain insights into program culture, trainee experiences, and educational environments that may not be easily conveyed through traditional program websites. The COVID-19 pandemic further accelerated the adoption of social media by both applicants and training programs, highlighting its growing role in recruitment and outreach. Gastroenterology fellowship programs are uniquely positioned to leverage these platforms given the visual and procedural nature of the specialty, which lends itself to digital content such as endoscopy demonstrations, educational discussions, and conference highlights. This editorial argues that gastroenterology fellowship programs should move beyond passive or reactive social media use toward a structured, intentional strategy, one built around 4 core pillars: recruitment transparency, educational dissemination, cultural representation, and inclusive outreach. We outline practical considerations including platform selection, content governance, and professionalism standards, while also acknowledging the risks of misrepresentation and inequity that programs must actively work to mitigate.

## INTRODUCTION

Social media has rapidly transformed communication, education, and professional networking across medical specialties, with graduate medical education programs increasingly adopting these platforms for recruitment and engagement.^1^ A systematic review found that X (formerly Twitter), podcasts, and blogs are frequently used to engage learners and enhance education in residency programs, while social media platforms are increasingly used to attract applicants.^1^ Multiple specialties have documented this trend: urology residency applicants reported widespread use of social media during the 2021 match, with more applicants than program directors feeling that social media helped them gain better insight into programs.^2^ Similarly, 62% of family medicine residency applicants used social media to research programs, with most expressing concern about obtaining sufficient information through virtual interviews alone.^3^ The COVID-19 pandemic accelerated this shift, with professional social media use among urology applicants increasing from 44% pre-pandemic to 80% in 2021.^4^

Fellowship applicants actively seek social media content when evaluating training programs. Across multiple specialties, applicants use these platforms to assess program culture, trainee lifestyle, and educational environment-factors difficult to capture through traditional websites.^2,3,5,6^ Among hematology-oncology fellowship applicants, 67% used X (formerly Twitter) and 70% used Instagram, with 55% acknowledging X (formerly Twitter) as a useful information source and 20% reporting that it influenced their program ranking decisions.^7^ In pediatric emergency medicine, 79% of applicants visited social media accounts of programs they applied to, though the influence on final ranking decisions varied.^5^ Orthopedic surgery applicants demonstrated particularly high engagement, with 61.9% using Instagram and 28.7% relying on it as their main resource when researching programs, with 60.6% reporting that social media positively impacted their perception of programs.^6^

Gastroenterology fellowship programs are uniquely positioned to benefit from strategic social media engagement. The procedural nature of the specialty lends itself to compelling visual content including endoscopic techniques, multidisciplinary tumor boards and liver procedure highlights that few other specialties can replicate as naturally. Yet despite this advantage, our field has lagged behind others in developing structures and purposeful digital strategies. Many program accounts remain inconsistent, unfocused, or entirely absent. We believe this represents a missed opportunity, not simply for recruitment, but for academic dissemination, community building and advancing equity in the fellowship application process.

The aim of this editorial was to provide gastroenterology fellowship programs with a practical opinionated framework organized around 4 pillars-for developing social media presence that is strategic, authentic, and ethically grounded.

## LAYING THE FOUNDATION: BUILDING A PROGRAM PRESENCE

Establishing a social media presence requires deliberate planning rather than spontaneous adoption. The American Academy of Neurology position statement emphasizes that simply signing up for a platform will not lead to success; programs must establish clear goals, define priorities, and ensure conveyance of intended messages.^8^ We propose organizing these goals around 4 core pillars: (i) recruitment transparency, (ii) educational dissemination, (iii) cultural representation, and (iv) inclusive outreach. Each subsequent section of this editorial maps onto 1 of these pillars.

Platform selection should align with applicant preferences and content strategy. Instagram has emerged as the preferred platform among many residency and fellowship applicants.^5,6^ X (formerly Twitter) serves as an effective platform for rapid information dissemination, academic discussion, and professional networking, with 67% of hematology-oncology applicants reporting its use.^7^ LinkedIn facilitates professional connections and faculty recruitment.^9^ For most gastroenterology fellowship programs starting from scratch, we recommend beginning with Instagram as the primary platform given its visual format, applicant reach, and relatively low barrier to consistent content creation. X (formerly Twitter) can then be layered in to amplify scholarly content and engage with the broader gastroenterology academic community. Programs should not feel compelled to maintain simultaneous active presence on every platform. Some cross-sectional studies demonstrate that larger programs with more fellowship positions are more likely to maintain active social media accounts ^10,11^

Defining account ownership and oversight is critical for sustainability and accountability. Programs must decide whether accounts will be managed by fellows, faculty, institutional communications teams, or a combination thereof. We recommend a comanagement model in which a dedicated fellow serves as primary content creator under the oversight of a faculty advisor who reviews posts before publication. This structure preserves authentic trainee voice—which applicants consistently find more credible than institutional messaging—while ensuring professionalism and Accreditation Council for Graduate Medical Education compliance.^12^ A rotating annual fellow role prevents burnout and ensures continuity. Accountability measures should include a scheduled posting calendar (targeting a minimum of 2–4 posts per week), quarterly review of engagement metrics such as follower growth, post reach, and interaction rates, and an annual content audit.^10,13^

Aligning content with institutional policies and professionalism standards is nonnegotiable. Programs must ensure that all content respects patient confidentiality, avoids sharing false information, and maintains professional boundaries.^8^ The American Medical Association and American College of Physicians have issued specific policies on social media use that emphasize maintaining professional conduct online.^8,14^ It is equally important to recognize that social media can inadvertently misrepresent program culture-a curated highlight reel of events and achievements may project an unrealistic or idealized image that sets false expectations for incoming fellows. Programs should strive for authenticity over polish and should develop written social media policies that address patient privacy, appropriate content, and consequences for unprofessional behavior, while providing education to fellows and faculty on digital professionalism.^1,8,12^

## PILLAR 1—RECRUITMENT TRANSPARENCY

Effective recruitment content provides prospective applicants with an honest, multidimension view of the fellowship experience. Studies demonstrate that applicants seek content reflecting trainee lifestyle, interpersonal relationships, and program culture, which can meaningfully influence perceptions of fellowship programs.^3,5,6^ Effective recruitment content includes visual and narrative posts showcasing day-to-day fellowship activities, procedural training opportunities, multidisciplinary conferences, and educational didactics.^6,13^ An analysis of hematology-oncology fellowship Instagram accounts revealed that posts highlighting “Fellow Life” emphasizing culture, wellbeing, camaraderie, and social activities—were significantly more frequent than those emphasizing medical education alone, suggesting programs recognize the value of cultural transparency.^15^ Video content introducing residents and faculty has been shown to generate the highest activity and reach.^13^ In practice, high-impact recruitment content includes fellow spotlight posts, “Day in the life” content, procedural highlights and event and environment posts (team dinners, wellness events and local city highlights).^3,5,16^

What distinguishes high-performing program accounts from lower-engagement ones is not production quality but consistency and authenticity. Accounts that post infrequently, rely primarily on generic infographics, or feature only senior faculty tend to underperform relative to accounts that post regularly, center fellow voices, and vary their content format. One general surgery program increased average post reach from 7 to over 4,500 people by shifting to personalized video content featuring residents and faculty^13^—a model directly applicable to gastroenterology fellowship programs.

## PILLAR 2—EDUCATIONAL DISSEMINATION

Social media extends the academic mission of fellowship programs beyond institutional walls. Platforms enable rapid sharing of research findings, visual abstracts, and conference highlights to a global audience.^8,9,17,18^ Practically, this can take the form of.

- Posting a custom visual abstract when a fellow or faculty member publishes a paper, tagging the journal and relevant professional societies to maximize reach.^19^
- Live-tweeting or posting during national GI meetings such as Digestive Disease Week (DDW), American College of Gastroenterology (ACG) meet, American Association for the Study of Liver Diseases (AASLD) to increase program visibility and engage the broader academic community.
- Sharing short educational reels on clinical topics—GI bleeding algorithms, IBD treatment ladders, hepatitis C screening updates-which build the program's reputation as an educational resource rather than simply a recruitment account.
- Featuring journal club summaries or interesting case discussions to demonstrate the program's intellectual culture.

The North American Society for Pediatric Gastroenterology, Hepatology and Nutrition has noted that institutional social media presence is independently associated with divisional ranking and reputation scores^9^-suggesting that consistent educational output has reputational benefits beyond recruitment alone. However, programs must ensure that educational content is accurate and properly vetted before publication. The rapid-dissemination nature of social media means that errors or oversimplifications spread quickly and can carry professional consequences. A faculty review step before posting clinical or educational content is strongly recommended.^8^

## PILLAR 3—CULTURAL REPRESENTATION

Program culture is among the most difficult qualities to convey through traditional websites, and social media is currently the most effective tool available for doing so. The goal is not to present a flawless version of the program, but to give prospective applicants a realistic and relatable picture of what it is actually like to train there. Previous studies have shown that applicants frequently seek social media content to assess the program culture, which can meaningfully influence perceptions of residency and fellowship programs.^20^

We believe programs should resist the temptation to manage their social media presence like a marketing campaign. Overly produced or uniformly positive content tends to feel institutional and inauthentic, and sophisticated applicants, who are themselves active social media users, can usually tell the difference. The most credible cultural signal a program can send is content created by current fellows, in their own voice, without excessive editorial smoothing. Posts that feature community engagement, cultural festivities, or the surrounding city environment may provide insight into the broader lifestyle associated with the program and can be particularly valuable for applicants who are unable to visit in person.^21,22^

At the same time, programs should be aware of the risk of amplifying a particular version of program culture that may not reflect the full diversity of trainee experiences. If social media content consistently features the same subset of fellows, it may inadvertently signal exclusion to applicants from underrepresented backgrounds. Intentional curation that includes a broad range of trainee voices is both an ethical obligation and a recruitment advantage.

## PILLAR 4—INCLUSIVE OUTREACH AND DIVERSITY, EQUITY, AND INCLUSION

Diversity, equity, and inclusion are the cornerstone of work environment in any institution, including healthcare. Data suggest persistent disparities across healthcare in terms of gender, race, and other sociodemographic factors.^23^ By sharing initiatives that promote inclusive mentorship, programs can demonstrate efforts to support trainees from diverse backgrounds and foster an environment where different perspectives are valued. Posts highlighting mentorship programs, outreach activities, and faculty or fellow achievements from underrepresented groups can help communicate a culture of inclusivity.^24^ In addition, social media can serve as a tool to promote equitable recruitment by increasing program visibility to a broader and more diverse pool of applicants, including those who may have limited access to traditional networking opportunities or institutional resources.

Programs must also recognize that social media itself is not an equitable playing field. Applicants with stronger existing professional networks, greater digital literacy, or more time to curate their own online presence may derive disproportionate benefit from social media-driven recruitment. Programs with larger budgets or dedicated communications staff will produce more polished content than smaller community programs. Acknowledging these structural asymmetries should prompt programs to use social media as a supplement to and not a replacement for direct outreach, holistic review, and structured diversity recruitment pipelines.

## FUTURE DIRECTIONS FOR SOCIAL MEDIA USE IN GASTROENTEROLOGY FELLOWSHIP PROGRAMS

As the role of social media in medical education continues to evolve, gastroenterology fellowship programs are well-positioned to lead several emerging applications. Multimedia-based educational content (short procedural videos, infographics, and visual abstracts) can enhance dissemination of complex clinical and technical knowledge in accessible formats.^25^^,^^26^ Social media may also facilitate genuine cross-program collaboration, including shared educational series, joint case conferences promoted across institutional accounts, and coordinated multicenter research promotion.

**Figure 1.**
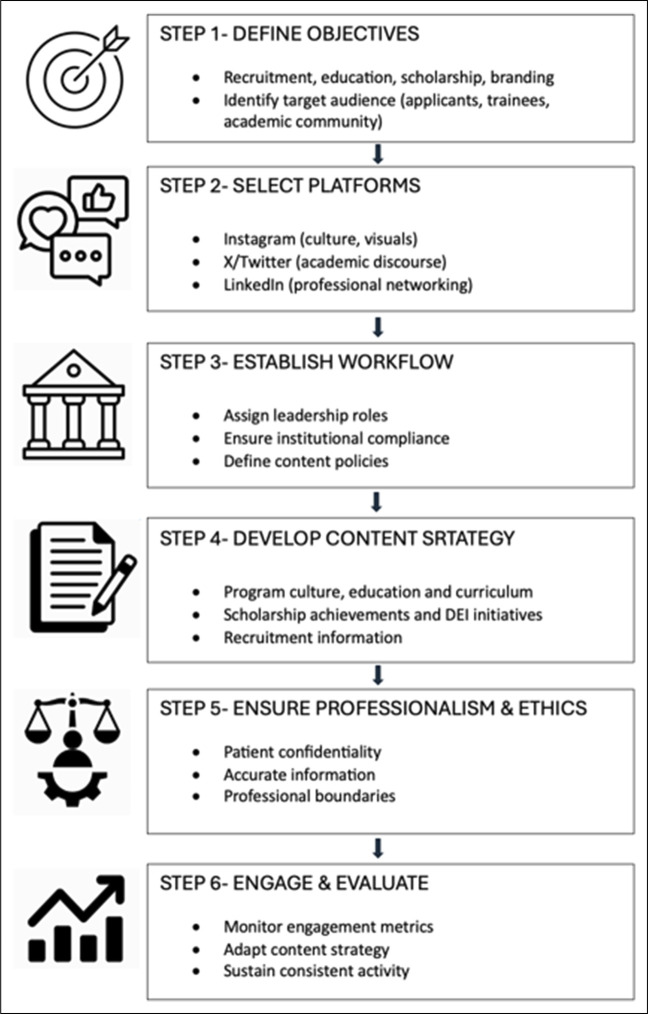
A stepwise framework outlining key components for establishing and maintaining a professional social media presence in gastroenterology fellowship programs.

As artificial intelligence tools increasingly enable automated content generation and scheduling, programs must be vigilant that AI-assisted social media does not erode the authenticity and human voice that make trainee-centered content effective in the first place. The goal of social media in fellowship recruitment is connection, not content volume. A flowchart depicting a practical framework is shown below (Figure 1)

## CONCLUSIONS

Social media is no longer optional for competitive gastroenterology fellowship programs-it is an expected channel through which applicants evaluate, compare, and form impressions of training environments. But presence alone is insufficient. What distinguishes programs that benefit from social media from those that merely participate is intentionality: clear goals, consistent execution, authentic fellow voices, and honest representation of program culture.

We recommend that program directors approach social media through the 4-pillar framework outlined here-recruitment transparency, educational dissemination, cultural representation, and inclusive outreach and that they designate a comanagement structure with protected fellow and faculty roles to sustain it. Programs should audit their current presence against these pillars and identify the single most underdeveloped area as a starting point for improvement.

Social media will not compensate for deficiencies in clinical training, mentorship, or program culture. But when those foundations are strong, a thoughtful social media strategy ensures that strength is visible to the applicants who would thrive in that environment and who might never have found it otherwise.

## DISCLOSURES

Author contributions: Substantial contributions to the conception or design of the work; or the acquisition, analysis, or interpretation of data for the work: J. Bahirwani, M. Changela, P. Kalyankar, H. Ali and DS Dahiya. Drafting the work or reviewing it critically for important intellectual content; final approval of the version to be published; agreement to be accountable for all aspects of the work in ensuring that questions related to the accuracy or integrity of any part of the work are appropriately investigated and resolved: All authors. DS Dahiya is the article guarantor.

Financial disclosure: None to report.

## ABBREVIATIONS:

AASLD: American Association for the Study of Liver Diseases
ACG: American College of Gastroenterology
ACGME: Accreditation Council for Graduate Medical Education
AI: artificial intelligence
DDW: digestive disease week
DEI: diversity, Equity and Inclusion
GI: gastroenterology
IBD: inflammatory bowel disease
